# Surface Dependent Dual Recognition of a G-quadruplex DNA With Neomycin-Intercalator Conjugates

**DOI:** 10.3389/fchem.2020.00060

**Published:** 2020-02-12

**Authors:** Nihar Ranjan, Katrine F. Andreasen, Yashaswina Arora, Liang Xue, Dev P. Arya

**Affiliations:** ^1^Laboratory for Medicinal Chemistry, Department of Chemistry, Clemson University, Clemson, SC, United States; ^2^Department of Medicinal Chemistry, National Institute of Pharmaceutical Education and Research, Raebareli, India

**Keywords:** G-quadruplex, *Oxytricha Nova*, neomycin, DNA, FID

## Abstract

G-quadruplexes have been characterized as structures of vital importance in the cellular functioning of several life forms. They have subsequently been established to serve as a therapeutic target of several diseases including cancer, HIV, tuberculosis and malaria. In this paper, we report the binding of aminosugar-intercalator conjugates with a well-studied anti-parallel G-quadruplex derived from *Oxytricha Nova* G-quadruplex DNA. Of the four neomycin-intercalator conjugates studied with varying surface areas, BQQ-neomycin conjugate displayed the best binding to this DNA G-quadruplex structure with an association constant of *K*_a_ = (1.01 ±0.03) × 10^7^ M^−1^ which is nearly 100-fold higher than the binding of neomycin to this quadruplex. The binding of BQQ-neomycin displays a binding stoichiometry of 1:1 indicating the presence of a single and unique binding site for this G-quadruplex. In contrast, the BQQ-neomycin displays very weak binding to the bacterial A-site rRNA sequence showing that BQQ-does not enhance the neomycin binding to its natural target, the bacterial rRNA A-site. The BQQ-neomycin conjugate is prone to aggregation even at low micromolar concentrations (4 μM) leading to some ambiguities in the analysis of thermal denaturation profiles. Circular dichroism experiments showed that binding of BQQ-neomycin conjugate causes some structural changes in the quadruplex while still maintaining the overall anti-parallel structure. Finally, the molecular docking experiments suggest that molecular surface plays an important role in the recognition of a second site on the G-quadruplex. Overall, these results show that molecules with more than one binding moieties can be made to specifically recognize G-quadruplexes with high affinities. The dual binding molecules comprise of quadruplex groove binding and intercalator units, and the molecular surface of the intercalator plays an important part in enhancing binding interaction to the G-quadruplex structure.

## Introduction

G-quadruplex structures are highly polymorphic DNA structures that can exist in dynamic equilibrium with other possible orientations of the same structure (Seenisamy et al., 2004). G-quadruplexes have been found at the telomeric ends and they have also been shown to form in several segments of the genomic DNA as well (Huppert and Balasubramanian, 2007). In several cases, the formation of G-quadruplex is only transient to propagate cellular functions and often they work in tandem with proteins (Wen and Gray, 2002; Hegyi, 2015). Because of the ability of G-quadruplex to serve as a central structure to control cell integrity and its role in transcriptional control, they have been regarded as a target for developing new therapeutics, especially for cancer (Neidle, 2010). However, several other life forms such as bacteria, fungus and viruses have been shown to possess regions capable of forming G-quadruplex and they have been suggested to play prominent roles in their life progression (Thakur et al., 2014; Artusi et al., 2015; Perrone et al., 2017). Due to these advances in the field, G-quadruplexes have moved far away from being a cancer-centric target to a structure which has relevance in the design of new antibacterial, antifungal and antiviral agents.

Formation of G-quadruplex requires stacked G-tetrads which are formed by the planar assembly of four guanines using eight Hoogsteen hydrogen bonds thus making these structures highly thermally stable (Burge et al., 2006). Each quadruplex discovered till date has unique distinguishing features. The uniqueness primarily lies in their distinct folding patterns. The differences in the folding patterns are inherent to changes in the loop connectivity and stabilizing metal cations resulting in large differences in the groove structures (Hazel et al., 2004; Bhattacharyya et al., 2016). Therefore, unlike the defined groove features of A-form and B-form DNA structures, quadruplex grooves show large variations in groove widths, depths, inter-phosphate distances between the DNA strands and base orientations which are ultimately related to the size of the stabilizing metal cations, as well as, the length, base composition and directionality of the loops. The differences in the groove widths and shapes offer new vistas for their recognition where dual recognition strategies can be employed given to the proven predilection of G-tetrads for chemical moieties containing planar fused polycyclic aromatic rings with extended π-systems (Monchaud and Teulade-Fichou, 2008).

**Figure 1:**
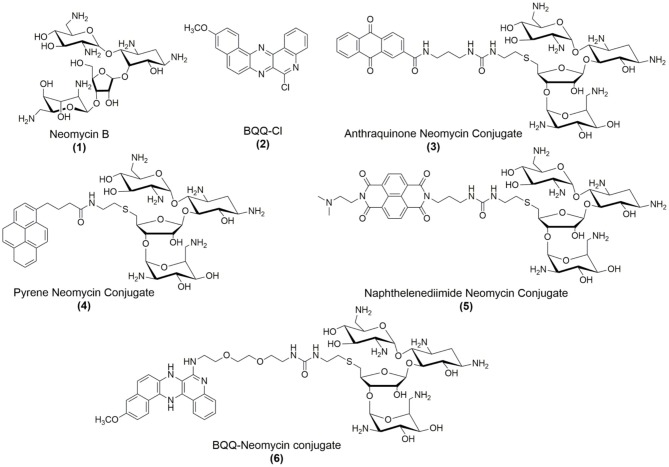
Chemical structures of compounds used in this study.

Using different recognition strategies, we have tailored the synthesis of dual and triple binding ligands to achieve molecular recognition of a variety of nucleic acid forms including duplexes, triplexes, quadruplexes and single-stranded nucleic acids and nucleic acid models of functionally relevant regions of DNA and RNA structures (Xue et al., 2002, 2011; Charles et al., 2007; Xi et al., 2009; Willis and Arya, 2010; Kumar and Arya, 2011; Ranjan and Arya, 2013, 2016; Ranjan et al., 2013a,b; Watkins et al., 2013, 2017; Kellish et al., 2014; Jiang et al., 2015; Nahar et al., 2015; Kumar et al., 2016; Degtyareva et al., 2017; Story et al., 2019). We have previously reported that neomycin (Figure 1), which is an antibiotic belonging to the aminoglycoside class, can recognize DNA G-quadruplex structures with moderate (Ranjan et al., 2010) to high affinities (Ka ~ 10^5^-10^8^ M^−1^) and its binding can be modulated by converting it into a dual binding ligand (Xue et al., 2011; Ranjan et al., 2013a). Conjugating neomycin with G-quadruplex binding moieties can lead to up to 1,000-fold enhancement in the binding affinity to target G-quadruplexes than the individual moieties (Ranjan et al., 2013a). Taking cues from these results, we wished to explore the effect of fused aromatic surfaces in the DNA G-quadruplex recognition. Accordingly, four neomycin-intercalator conjugates (Figure 1) that differed in the ring size and composition of the intercalator were taken to study the effect of the stacking surfaces in the G-quadruplex recognition. For the G-quadruplex, we chose the telomeric G-quadruplex derived from *Oxytricha Nova* which is a well-resolved (Schultze et al., 1994), structurally well-understood and well-defined DNA quadruplex structure that adopts anti-parallel orientation (Smith and Feigon, 1992). Moreover, given our prior understating of neomycin binding to the G-quadruplex DNA structure (Ranjan et al., 2010), we chose this sequence to understand the π-surface area and composition effects in the dual recognition of G-quadruplexes.

In the presence of sodium salt, *Oxytricha Nova* telomeric DNA adopts an antiparallel G-quadruplex structure with four grooves of widths ~6–18 Ǻ (Schultze et al., 1994). The four grooves, formed in this quadruplex are potential binding sites of aminoglycosides. In this report, we sought to understand the effect of ring size and composition on G-quadruplex dual recognition in which neomycin was presumed to play the anchor role. To see the relevance of these conjugates in nucleic acid selectivity, we have compared the binding of the best binder with a duplex DNA and the bacterial rRNA A-site which is the natural and high affinity target of aminoglycosides (Arya, 2007).

## Materials and Methods

### Nucleic Acid Samples

The DNA oligonucleotides samples used in this study were purchased from IDT (Coraville, IA). The RNA oligonucleotides were purchased from Dharmacon Research. All nucleic acid samples were used without further purification. The concentrations of both DNA and RNA sample solutions were determined by measuring UV absorbance at 85°C to ensure that the nucleic acids did not possess any secondary structure. The DNA quadruplex structure was formed by heating the stock nucleotide solution in a buffer solution containing sodium ions (Na^+^, 100 mM), to 95°C for 15 min and then slowly cooling the samples back to room temperature in a non-controlled manner. The DNA quadruplex samples were then left to incubate at 4°C for 2 days before they were used for further experiments. The DNA duplex sample was formed by heating it to 95°C for 5 min in a buffer containing 10 mM sodium cacodylate, 0.5 mM EDTA and 100 mM NaCl at pH 7.0. The sample was brought to room temperature by allowing it to self-cool and then incubating it at 4°C overnight before it was used for experiments. The bacterial rRNA A-site oligonucleotide sample was heated to 95°C for 5 min in a buffer containing 10 mM sodium cacodylate, 0.5 mM EDTA, and 100 mM NaCl at pH 7.0 and then it was quickly snap-cooled on ice. It was then stored at 4°C overnight before it was used for further experiments.

### Chemicals

Aminoglycosides used in the study were purchased from MP Biomedicals (Solon, OH). All chemicals were used without further purification. The neomycin conjugates used in this study were prepared in-house (Xue et al., 2010).

### UV-vis Spectroscopy

The thermal denaturation experiments were obtained on a Cary 100 UV-vis spectrophotometer containing a multi-cell (12 cell) holder equipped with pettier thermal controller. For all experiments, Quartz cells with 1 cm path length were used. The spectrophotometer stability was checked prior to each melting point experiment. The DNA melting was monitored at 260, 235, and 295 nm wavelengths and the samples were denatured between 20 and 98°C at the heating rate of 0.2°C/min. Data points were recorded every 1.0°C. The resulting temperature-absorbance plots were plotted using Kaleidagraph 3.5 software. The melting temperatures were determined as described by Mergny (Mergny and Lacroix, 2003).

### Circular Dichroism Experiments

The circular dichroism (CD) experiments were performed using a JASCO J-810 spectropolarimeter containing a four cell multi-cell holder and equipped with thermo-electrically controlled Chiller. Each CD spectrum was recorded as an average of three scans at 20°C. For CD titration experiments, a concentrated solution of the ligand was serially added to the DNA quadruplex sample and allowed to equilibrate for 5 min before a scan was taken. The resulting scan was plotted for CD signal changes with respect to wavelength or with respect to the number of equivalents taken to get the stoichiometry details.

### Fluorescence Intercalator Displacement (FID) Studies

The fluorescence intercalator displacement experiments reported in this study were performed on a Photon Technology International instrument (Lawrenceville, NJ) equipped with a temperature controller. All experiments reported in this study were performed at 20°C. The experiments were performed in a 3 mL quartz cell having a path length of 10 mm. The concentration of nucleic acid solutions used in this study was 0.25 μM/quadruplex or duplex or strand (for bacterial rRNA A-site studies). For each FID experiment the nucleic acid sample was mixed with Thiazole orange (TO) with a concentration of 0.50 μM. All experiments reported in this study were performed in 10 mM sodium cacodylate, 0.5 mM EDTA and 100 mM NaCl at buffer pH 7.0. The ligands were then serially added to the DNA/TO solution until the TO fluorescence was completely quenched. Each ligand addition was followed by a 4 min equilibration time before the fluorescence spectrum was recorded. The TO excitation was at 501 nm and the emission was then recorded from 510 to 650 nm. In all the experiments, the excitation and emission slit widths were 1.5 mm. For determination of binding constant, Scatchard analysis was done using the procedure described previously by Boger et al. (2001) in which—

(1) $$\begin{array}{l} \left(\Delta {\text{F}}_{\text{X}}/\Delta {\text{F}}_{\text{Sat}}\right).\left(1/\text{X}\right)=\text{Fraction of DNA}-\text{ligand Complex} \end{array}$$

(2) $$\begin{array}{l} \left[1-\left(\text{Δ}{\text{F}}_{\text{X}}/\Delta {\text{F}}_{\text{Sat}}\right).\left(1/\text{X}\right)\right]=\text{Fraction of free ligand} \end{array}$$

(3) $$\begin{array}{l} {\left[\text{DNA}\right]}_{\text{T}}.\left[\text{X}-\left(\Delta {\text{F}}_{\text{X}}/\Delta {\text{F}}_{\text{Sat}}\right)\right]=\left[\text{Free ligand}\right] \end{array}$$

Where,

*X* = Molar equivalent of ligand to quadruplex DNA
ΔF_X_ = Change in fluorescence
ΔF_Sat_ = Change in fluorescence when the quadruplex DNA is fully saturated with ligand
[DNA]_T_ = Total DNA concentration.

### Aggregation Studies

The aggregation behavior of BQQ-neomycin conjugate was checked using the Cary 100 UV-vis spectrophotometer. Both thermal melting and concentration-dependent absorbance studies were done to check the aggregation behavior. In thermal melting studies, the BQQ-neomycin conjugate was heated in 10 mM sodium cacodylate, 0.5 mM EDTA and 100 mM NaCl at buffer pH 7.0 at 2.5 and 5.0 μM concentration between 30 and 98°C and resulting absorbance vs. temperature graph was plotted. For concentration-dependent absorbance studies, different concentrations (0.2–36 μM) of the BQQ-neomycin conjugate were scanned between 200 and 600 nm and the resulting absorbance vs. concentration graph was plotted. The experiments were done in buffer 10 mM sodium cacodylate, 0.5 mM EDTA and 100 mM NaCl at buffer pH 7.0 at 23°C.

### Molecular Docking Studies

All docking experiments were performed as blind dockings where the blind docking refers to the use of a grid box which is large enough to cover all possible ligand-receptor interactions. AutoDock Vina, which has several advantages over its preceding version AutoDock 4.2, was used to perform docking experiments. The G-quadruplex receptor structure was obtained from Protein Data Bank (PDB ID: 156D) which was used for docking experiments without any further modification in the structure. The ligand structures were created using ChemDraw 14 and then brought to their energetically minimized structures using the Vega ZZ program (Pedretti et al., 2004) utilizing a conjugate gradient method with SP4 force field. Autodock tools version 1.5.6 was used to convert the ligand and receptor molecules to the proper file formats for docking. The docking experiments were performed using an exhaustiveness value 50. All other parameters were used as defaults. All rotatable bonds within the ligand were allowed to rotate freely, and the receptor was considered rigid. The final rendering of the docked structures was done using PyMol (www.pymol.org).

## Results and Discussion

### Fluorescence Intercalator Displacement Experiments

Fluorescence intercalator displacement assay (FID) was developed in Boger's laboratory (Boger and Tse, 2001; Boger et al., 2001; Tse and Boger, 2004) allowing for the screening of a variety of structurally and chemically diverse set of ligands. Unlike some other ligand screening methods which mandate the presence of a fluorophore in the ligand to be screened, this assay can be used to screen both fluorescent as well as non-fluorescent molecules binding to the nucleic acids. The utility of this assay has been demonstrated by many laboratories which have used both canonical and non-canonical nucleic acid structures of biological interest (Monchaud et al., 2006; Kumar and Arya, 2011; Kumar et al., 2011, 2015; Kellish et al., 2014). More importantly several applications of this assay have been shown to be useful in discovering G-quadruplex binding ligands (Monchaud et al., 2006).

In essence, this assay exploits the fluorescence enhancement of nucleic acid binding dyes that show non-specific binding and moderate affinities to their cognate structures. The intercalating dyes such as ethidium bromide or Thiazole orange are traditional intercalators used in this assay but recently the use of dyes such as Thioflavin T (ThT) is gaining momentum due to its special liking for certain G-quadruplex structures (Mohanty et al., 2012; Verma et al., 2019). In a typical experiment, the nucleic acid of interest is mixed with the intercalating dye in an appropriate ratio. The ratio of nucleic acid to the intercalator to be used in this assay is determined usually by running a fluorescence titration experiment in which the nucleic acid of interest is titrated against the intercalator ligand and the resulting fluorescence changes are used to construct a plot giving the stoichiometry of the intercalator-nucleic acid interaction. The FID assay is thus developed in which the ligand library to be screened is titrated into a solution containing the nucleic acid and the intercalator at a fixed ratio.

In our FID experiments, we used Thiazole orange (TO) which is much more responsive to fluorescence emission in comparison to the other popularly used intercalator: ethidium bromide (Boger et al., 2001). Thiazole orange binds to the *Oxytricha Nova* G-quadruplex DNA with a stoichiometry of 2:1 and for that reason, we took the same ratio of receptor to intercalator in our FID experiments. Addition of different ligands to the G-quadruplex-intercalator complex caused successive decrease in the fluorescence signal till the fluorescence signal reached the level of Thiazole orange fluorescence emission in the absence of the G-quadruplex DNA.

**Figure 2:**
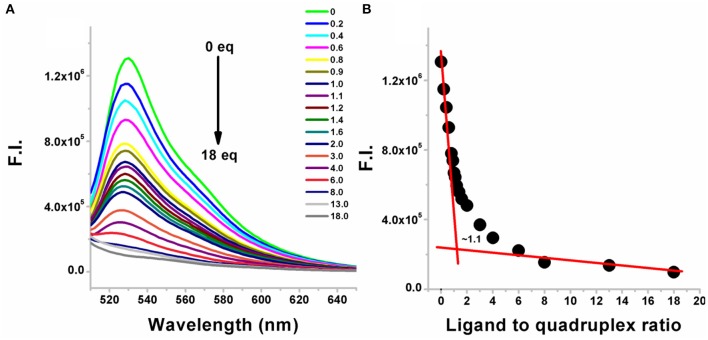
**(A)** Fluorescence emission changes in the G-quadruplex-thiazole orange complex upon addition of neomycin-BQQ conjugate (compound **6**). The number of equivalents of compound **6** added from 0 to 18 equivalents is shown for each point. **(B)** The binding stoichiometry plot showing the binding stoichiometry of compound **6** with the *Oxytricha Nova* quadruplex. The experiments were performed in buffer 10 mM sodium cacodylate, 0.5 mM EDTA, 100 mM NaCl at pH 7.0 (*T* = 20°C). Thiazole orange was excited at 501 nm and the emission spectrum was recorded between 510 and 650 nm.

A representative example is shown in Figure 2A in which BQQ-neomycin conjugate (compound 6) was successively added to *Oxytricha Nova*-TO complex. As increasing amounts of compound **6** were added, the fluorescence emission intensity of TO continued to decrease suggesting displacement of bound Thiazole orange. The addition of ligand was continued till no more changes in the intensity of the TO emission signal was observed suggesting complete displacement of the bound TO. Such TO displacement titrations can also be used to determine the binding stoichiometry by plotting the percent fluorescent change with respect to number of the equivalents of the ligand added. As shown in Figure 2B, the displacement assay revealed that BQQ-neomycin conjugate (compound **6**) binds to the *Oxytricha Nova* G-quadruplex in 1:1 ratio. The resulting displacement pattern was then plotted to obtain DC_50_ values where DC_50_ refers to the ligand concentration required to displace half of the bound intercalator from the G-quadruplex in these assays.

As shown in Table 1, Figure S1, DC_50_ values were calculated for different neomycin conjugates (Figure 1) along with the control ligands. Among the intercalators studied, BQQ-neomycin conjugate (compound **6**) showed the least amount of ligand (422-fold lesser than neomycin) required to displace 50% intercalator followed by anthraquinone-neomycin conjugate (253-fold lesser than neomycin). The other neomycin-intercalator conjugates studied, pyrene-neomycin (compound **4**) and naphthalenediimide-neomycin (compound **5**), also showed superior DC_50_values than neomycin (16 and 10-folds lower, respectively). As a control, we performed the DC_50_ determination of a control duplex sequence d(AGGGGTTTTCCCCT) (Table 1, Figure S2). The DC_50_ values obtained for this experiment showed that the preference for quadruplex for BQQ-neomycin conjugate (compound **6**) was at least 3.5-fold higher while the same for pyrene-neomycin (compound **4**) was 2-fold higher than the duplex DNA. The other neomycin intercalators conjugates showed DC_50_ values lower for duplexes. These results clearly established that neomycin-anthraquinone and neomycin-BQQ conjugates preferentially bind to *Oxytricha Nova* G-quadruplex in comparison to a duplex DNA sequence whereas for neomycin pyrene and neomycin naphthalenediimide conjugates preference for the DNA duplex was more. Therefore, we turned our attention to the best quadruplex binder of the compounds studied, the neomycin-BQQ conjugate (compound **6**) for further detailed studies.

**Table 1:** A table listing the DC_50_ values for different neomycin intercalator conjugates (compounds 1–6) with *Oxytricha Nova* G-quadruplex DNA and a DNA duplex sequence.

**Ligand**	**d(GGGGTTTTGGGG) DC_50_ (μM)**	**d(AGGGGTTTTCCCCT) DC_50_ (μM)**
Neomycin	76.02	53.44
BQQ-Cl	>250	–
Neomycin- Pyrene	4.73	2.61
Neomycin-Anthraquinone	0.30	0.75
Neomycin-BQQ	0.18	0.64
Neomycin-Naphthalenediimide	7.89	0.83

### Determination of Association Constant of BQQ-Neomycin Conjugate

**Figure 3:**
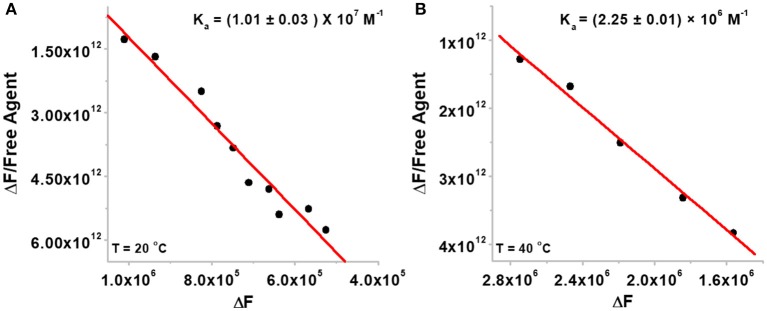
The Scatchard plots of BQQ-neomycin binding to *Oxytricha Nova* G-quadruplex DNA at **(A)** 20°C and **(B)** 40°C. The experiments were performed in buffer 10 mM sodium cacodylate, 0.5 mM EDTA, 100 mM NaCl at pH 7.0.

Since neomycin-BQQ conjugate binds to *Oxytricha Nova* G-quadruplex DNA sequence in a 1:1 ratio (Figure 2B), we performed a Scatchard analysis of the BQQ-neomycin FID titration data as described by Boger et al. (2001) and Yeung et al. (2003). The analysis (Figure 3) revealed that BQQ-neomycin conjugate (compound **6**) binds to the *Oxytricha Nova* G-quadruplex sequence with an affinity of *K*_a_ = (1.01 ± 0.03) × 10^7^ M^−1^. We have previously shown that under same conditions the association constant (K_a_) of neomycin for *Oxytricha Nova* sequence was (1.03 ± 0.50) × 10^5^ M^−1^ (Ranjan et al., 2010). This result shows that conjugation of neomycin to BQQ leads to 98-fold higher affinity for the quadruplex. However, the affinity of BQQ-neomycin was found to be temperature-dependent as increasing the study temperature to 40°C led to nearly a 4-fold decrease in the affinity of the conjugate with a *K*_a_ = (2.25 ± 0.01) × 10^6^ M^−1^.

### Comparative Binding With the Bacterial A-Site RNA

**Figure 4:**
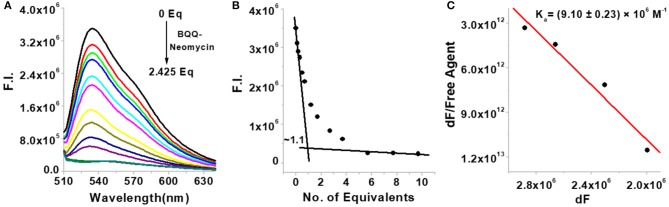
**(A)** FID titration of bacterial rRNA A-site with BQQ-neomycin conjugate **(B)** binding stoichiometry plot of BQQ-neomycin conjugate's interaction with the rRNA A-site. **(C)** Scatchard analysis of binding interaction. The concentration of rRNA A-site was 0.25 μM and the experiments were performed in buffer 10 mM sodium cacodylate, 0.5 mM EDTA, 100 mM NaCl at pH 7.0 at 20°C.

Since the bacterial rRNA A-site is the natural target of aminoglycosides, we investigated how the conjugation of neomycin affects the binding of neomycin to the bacterial rRNA A-site and how does the overall binding of BQQ-neomycin compare to all of its possible binding targets. As shown in Figure 4A, the addition of BQQ-neomycin conjugate to the bacterial rRNA-TO complex led to a continuous decrease in the fluorescence emission of TO. The FID titration was stopped when further addition of BQQ- neomycin led to negligible changes in the emission signal of TO. Further analysis of the binding event showed that like the *Oxytricha Nova* G-quadruplex DNA, it binds with the rRNA A-site sequence with 1:1 binding stoichiometry (Figure 4B). Scatchard analysis (Figure 4C) revealed that BQQ-neomycin binds with the rRNA A-site with an affinity of *K*_a_ = (9.10 ± 0.23) × 10^6^ M^−1^ which is lower than the binding of the same with the *Oxytricha Nova* G-quadruplex sequence. Further analysis of the results showed that the DC_50_ value for this FID experiment was (276.0 ± 8.2) nM while the same for neomycin was (135.4 ± 9.4) nM. These results showed that conjugation of BQQ to neomycin makes it a poor bacterial rRNA A-site binder by nearly 2-fold change in the affinity.

### CD Experiments Show Evidence of Complexation of *Oxytricha nova* Quadruplex With the BQQ-Neomycin Conjugate

**Figure 5:**
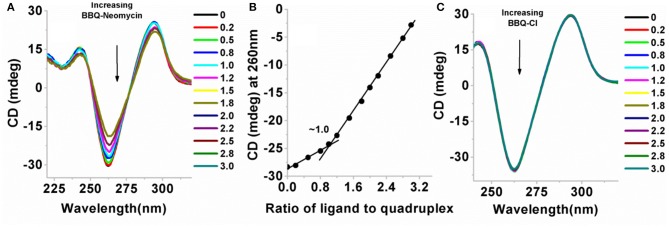
**(A)** CD titration plot of BQQ-neomycin with the *Oxytricha Nova* G-quadruplex DNA. The ratios of BQQ-neomycin to the quadruplex are indicated in the legend. **(B)** CD detected binding stoichiometry plot of BQQ-neomycin interacting with the *Oxytricha Nova* G-quadruplex DNA. **(C)** Control titration experiment of BQQ-Cl with the *Oxytricha Nova* G-quadruplex DNA. The DNA concentration used in the experiments was 10 μM/strand and both the experiments were performed in buffer 10 mM sodium cacodylate, 0.5 mM EDTA, 100 mM NaCl at pH 7.0 at 20°C. Small aliquots of ligand were serially added [as indicated on plots **(A,C)**] to the DNA solution. During the titration, the DNA solution was stirred with the added ligand for 5 min followed by another 5 min of equilibration time. The CD signal represents an average of three scans.

Circular dichroism experiments have been extensively used for the study of G-quadruplexes (Randazzo et al., 2012; Vorlíčková et al., 2012). The different types of G-quadruplexes show unique CD signature peaks in which parallel quadruplexes show absorption maximum at 260 nm and the minimum at 240 nm, while the antiparallel quadruplexes show an absorption maxima at 295 nm and the minimum is observed at 260 nm (Balagurumoorthy and Brahmachari, 1994). We performed CD titrations to find out (a) the ligand-induced changes in the CD signal to observe any conformational change accompanying the ligand binding, and (b) to find out the binding site size for the ligand to quadruplex. The CD titration of the *Oxytricha nova* quadruplex was initially performed with the BQQ-neomycin conjugate (Figure 5A). In the absence of ligand, the maximum of the CD signal was observed at ~295 nm while the minimum was at ~260 nm which was consistent with the antiparallel structure of this G-quadruplex as reported previously (Balagurumoorthy et al., 1992).

When BQQ-neomycin was added to the *Oxytricha Nova* quadruplex, significant changes in the CD signal were observed (Figure 5A). With the addition of small aliquots of the ligand, the positive CD signal at 295 nm continuously diminished. At high ligand to quadruplex ratios (more than three equivalents of ligand to the DNA), DNA precipitation was observed. Concurrently, the CD signal at 260 nm became much more negative upon addition of incremental amounts of ligand. From this titration, it is sufficiently evident that the mode of interaction of BQQ-neomycin conjugate is very different from neomycin which produces no change in the CD signal of the quadruplex even at much higher ligand concentrations than what has been used in this experiment. This reaction also confirms complexation of BQQ-neomycin to the *Oxytricha nova* G-quadruplex DNA. Analysis of the CD signal changes with respect to the ligand ratios added yielded the binding stoichiometry plot which showed 1:1 binding of the BQQ-neomycin conjugate with the *Oxytricha nova* G-quadruplex (Figure 5B). The binding of BQQ-Cl did not lead to any observable change in the CD signal (Figure 5C), which suggests that in the case of BQQ-neomycin titration, both neomycin and the BQQ moieties bind synergistically with the G-quadruplex. A series of compounds which are structurally similar to BQQ have shown quadruplex binding properties (Hounsou et al., 2007). These compounds have been proposed to have π-stacking interactions with the guanine bases. Another example where neomycin has been conjugated to such planar aromatic compounds has also been reported (Kaiser et al., 2006). It has been proposed that the planar aromatic part in this molecule also involves π- stacking interactions with the G-tetrad and neomycin has a role in establishing this conjugate as a G-quadruplex specific ligand (Kaiser et al., 2006). Altogether, the results obtained with CD experiments are in agreement with the previously reported findings.

### UV Thermal Melting Experiments

**Figure 6:**
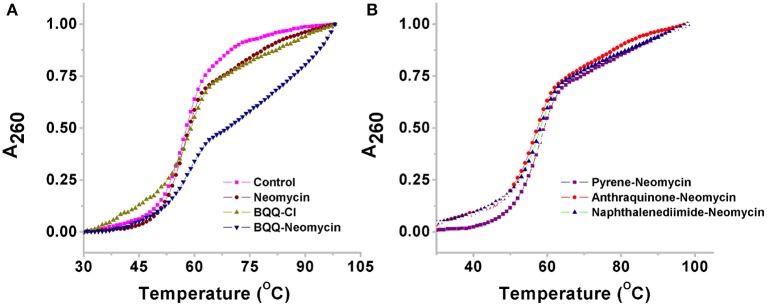
Thermal denaturation profiles of **(A)** *Oxytricha Nova* G-quadruplex DNA in the absence and presence of BQQ-neomycin conjugate and its constituent ligand neomycin and BQQ-Cl. **(B)** *Oxytricha Nova* G-quadruplex DNA in the presence of pyrene-neomycin, anthraquinone neomycin and naphthalenediimide-neomycin conjugates. The experiments were performed at 10 μM/Strand (5 μM in quadruplex) DNA concentration and the ligands were added at 1:1 quadruplex to ligand ratio. The heating was done at a rate of 0.2°C per minute. All experiments were in buffer 10 mM sodium cacodylate, 0.5 mM EDTA, 100 mM NaCl at pH 7.0 and the absorbance data was normalized for comparison.

UV monitored thermal denaturation experiments of nucleic acids are routinely used to study ligand-biomolecule interactions. The thermal melting experiments were performed to find any ligand-induced thermal stabilization by doing experiments both in the absence and presence of ligands. We monitored the changes in absorbance with respect to temperature at both 260 and 295 nm wavelengths. While in most quadruplexes the changes in absorbance are more prominent at 295 nm as compared to 260 nm (Mergny et al., 1998), we got satisfactory thermal melting profiles at 260 nm as shown in Figure 6. The *Oxytricha nova* quadruplex melted at 56.9°C in the absence of any ligand (Figure 6A). In the presence of pyrene-neomycin, anthraquinone neomycin, naphthalenediimide-neomycin (Figure 6B) or BQQ-Cl and neomycin alone, appreciable thermal stabilization was not observed (Table 2). However, in the presence of BQQ-neomycin, we saw signs of thermal stabilization of the G-quadruplex DNA as a biphasic thermal denaturation curve was obtained (Figure 6A). The first transition was observed at 59.8°C which is nearly three degree Celsius stabilization of the G-quadruplex. However, soon after the end of the first transition, a second transition begins which is much broader and with a middle point at 82.0°C. Such biphasic thermal denaturation profiles are usually observed when more than one nucleic acid species are involved in the melting process or different sets of complex events are taking place during the ligand-biomolecule interaction. Initially, we thought of higher temperature transition to be the result of thermal stabilization of the quadruplex by this ligand. However, such transition was completely missing when we carried out the CD melting experiment which showed only one transition belonging to the melting of the quadruplex (data not shown). Since molecules having polycyclic aromatic rings with extended π-conjugation are prone to self-stacking in polar media, we decided to perform further experiments to trace the origins of the second transition and study the aggregation behavior of BQQ-neomycin conjugate itself as discussed in the next section.

**Table 2:** UV detected thermal denaturation temperatures of *Oxytricha Nova* G-quadruplex DNA in the presence of various ligands.

**Ligand**	**Melting temperatures**	
	**T_m_**	**ΔT_m_ (in °C)**
None	56.9	–
Neomycin	56.9	0.0
BQQ-Cl	56.9	0.0
Pyrene-neomycin	58.8	1.9
Anthraquninone-neomycin	57.4	0.5
Naphthalenediimide-neomycin	57.5	0.6
BQQ-neomycin	59.8, 82.0	2.9^*^

* *Melting transition was masked by the ligand self-dissociation events*.

### Aggregation Studies of BQQ-Neomycin Conjugate

**Figure 7:**
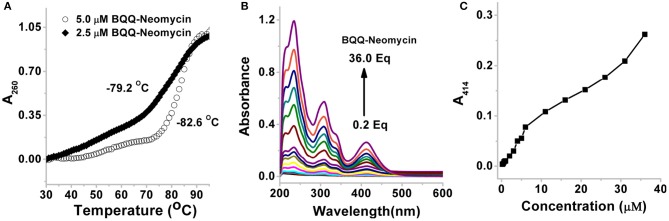
**(A)** Thermal denaturation profile of BQQ-neomycin conjugate at 2.0 and 5.0 μM concentrations in buffer 10 mM sodium cacodylate, 0.5 mM EDTA, 100 mM NaCl at pH 7.0. No DNA was added in these experiments. **(B)** UV-vis absorption spectra of BQQ-neomycin conjugate at increasing concentrations (0.2–36.0 μM). **(C)** Absorbance vs. concentration plot of BQQ-neomycin. All experiments were performed in buffer 10 mM sodium cacodylate, 0.5 mM EDTA, 100 mM NaCl at pH 7.0. The absorbance spectra was recorded at room temperature (23°C).

As shown in Figure 7A, the thermal melting of BQQ-neomycin conjugate was studied in the absence of DNA. In the absence of any DNA, the transitions that are observed are purely the resultant of temperature-dependent hyperchroism shown by the ligand itself. When the BQQ-neomycin conjugate was melted at a concentration of 2.5 μM, a slow rise in the absorbance of BQQ-neomycin conjugate was observed up to 70°C which abruptly rose afterwards. The overall melting profile appears similar to the one usually obtained in the presence of DNA. We performed another thermal denaturation experiment of BQQ-neomycin conjugate at 5.0 μM concentration. The denaturation profile in this experiment showed a much clearer melting transition with a T_m_ of 82.6°C. These experiments showed that BQQ-neomycin itself can show DNA like melting transitions and, therefore, caution must be taken in the interpretation of thermal denaturation of DNA-ligand complexes as ligands capable of self-stacking may mask, complicate or give misleading thermal melting temperatures. Such transitions can be attributed to the destacking of the ligand at higher temperatures as shown previously with Hoechst 33258 binding with the DNA (Kaushik and Kukreti, 2003).

We, then, sought to understand the concentration dependence of aggregation of BQQ-neomycin conjugate. As shown in Figure 7B, the absorbance spectra of BQQ-neomycin was recorded from low (0.2 μM) to moderate (36.0 μM) micromolar concentrations. The absorbance peak at 414 nm was then used to plot absorbance vs. concentration plot (Figure 7C) which clearly shows that the loss in the linearity of the graph is easily discernible. The deviation in the linearity was detected early at 4.0 μM concentration which became more prominent at concentrations 5.0 μM and above clearly mirroring the results obtained with the thermal denaturation experiments. Overall, these results suggest that BQQ-neomycin conjugate is prone to aggregation in buffer solutions as used in these experiments.

### Molecular Docking Studies

**Figure 8:**
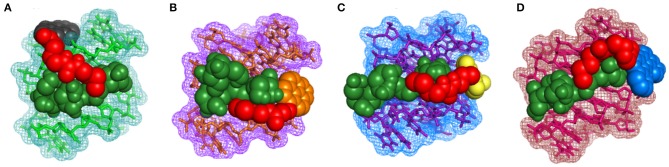
Lowest energy docked structures of various neomycin-intercalator conjugates with the *Oxytricha Nova* structure **(A)** Anthraquinone-neomycin **(B)** Pyrene-neomycin **(C)** Naphthalenediimide-neomycin **(D)** BQQ-neomycin. In each figure neomycin, linker and the Intercalator moieties have been shown in different colors.

To gain further insights into the molecular interactions taking place during quadruplex recognition, we performed docking studies with all four neomycin-intercalator conjugates (compounds **3–6**). AutoDock Vina was chosen to perform docking experiments because of (a) its ability to improve the average accuracy of binding mode predictions (b) its ability to take advantage of multiple core processors which significantly shortens the running time (c) its more efficient search of potential energy surfaces and (d) its high accuracy with ligands possessing more than 20 rotatable bonds. The structure used in the docking studies was retrieved from the protein data bank (PDB ID: 156 D) which is a refined NMR solution structure of the telomeric sequence d(GGGGTTTTGGGG) (Schultze et al., 1994). This G-quadruplex has been shown to adopt an antiparallel structure with the thymine loops occupying the opposite ends in the presence of sodium salt. The G-quadruplex formed from this telomeric structure gives rise to four distinct quadruplex grooves of which two are of medium groove widths (~12 Angstrom), one wide (~17 Angstrom) and one narrow (~6 Angstrom). We have previously shown that neomycin binds in the wide groove of this quadruplex as evidenced from solution NMR and molecular docking studies. The lowest energy docked structures for all four conjugates show that neomycin is positioned in the wide groove with linker extending the intercalating moieties more toward the thymine loop regions possibly to avoid steric clashes in the groove and also because of more vacant space available around thymine loops (Figure 8). BQQ-neomycin, naphthalenediimide neomycin and pyrene neomycin displayed nearly similar mode of binding in which the neomycin and the linker cover majority of the wide groove while the intercalator part of the molecule protrudes away toward the thymine. Despite this semblance in recognition, neomycin adopts different poses in all these structures to reorganize its rings in order to maximize the binding interactions. The predominant binding interaction involves hydrogen bonds, stacking and van der Waals interactions in all these interactions. The anthraquinone-neomycin conjugate displayed a strikingly different result in which the anthraquinone moiety moves out of the wide groove and then reaches out directly to the originating 5′-guanine base on one of the strands and makes stacking interactions with the guanine base. Although, similar behavior would be expected from all other intercalator moieties used in this study, the difference could likely be the outcome of the ring size of the intercalator units used. Of the four intercalator moieties used in these studies, anthraquinone is the smallest of all with three fused rings whereas pyrene and naphthalenediimide have four rings each. BQQ has the largest intercalator part with five rings fused together. Because of these differences, the smallest anthraquinone moiety finds enough space to stack with the guanine while the other intercalator units face steric clashes to make such stacking contacts. Rather, their interactions are assisted through greater van der walls interaction in addition to the hydrogen bonding contacts made by the neomycin and linker units. To check if the variations in the linker composition had any effect on the binding poses displayed by these conjugates, we also ran additional set of docking experiments using the same linker as used in the case of Neomycin-BQQ conjugate. These docking experiments showed almost identical binding poses (Supporting Information, Figure S3) as obtained previously showing that linker composition didn't have any profound effect on the binding of the conjugates presented here. In summary, the intercalator moiety's total surface areas, nature of atoms present on them (hydrogen bonding donor or acceptor) and the ring size appear to be the prime determinants of these interactions where a smaller interacting may likely interact directly through the π-stacking interactions with the guanine bases.

### Interaction of BQQ-Neomycin With Other Nucleic Acids

In this section, we present the results of our previously reported BQQ-neomycin conjugate which varied slightly in the linker composition than the BQQ-neomycin presented in this article. Previously, a Neomycin-BQQ conjugate, which contained an oxygen free thiourea bearing linker, was studied for its binding to a variety of nucleic acids including an antiparallel human telomeric G-quadruplex DNA. Neomycin and BQQ are the two binding moieties which are known for their liking for triplex nucleic acid structures (Arya et al., 2001, 2003). BQQ-neomycin conjugate was found to be one of the best triplex binding agents known as yet (Arya et al., 2003). The results obtained using competition dialysis have shown that BQQ-neomycin's preference was highest for an RNA triplex followed by a DNA triplex in a quadruplex free assay. In case of a DNA triplex, the binding constant of BQQ-neomycin was *K*_a_ = 2.7 × 10^8^ M^−1^ (Xue et al., 2010). For the human telomeric quadruplex, the fluorescent intercalator displacement assay showed that BQQ-neomycin conjugate's liking was behind the anthraquinone-neomycin, pyrene neomycin and napthalenediimide-neomycin (Ranjan et al., 2013a). The binding constant obtained with the best binder anthraquinone-neomycin (Ranjan et al., 2013a) was *K*_a_ = 1.25 × 10^7^ M^−1^, a value which was very close the value of the same obtained with BQQ-neomycin used in this study (*K*_a_ = 1.01 × 10^7^ M^−1^). This result shows that quadruplex topoplogy plays an important role in its binding to different ligands. Overall the results demonstrate that nucleic acid systems providing large surface areas for interactions, such as quadruplexes and triplexes are preferred nucleic acids for BQQ-neomycin conjugates where its binding affinity is in the range of *K*_a_ ~10^7^-10^8^ M^−1^.

## Conclusions

The ability of ligands to recognize G-quadruplex DNA structures has been shown to impede with the functions of telomerase. The core aspect of this inhibition lies in the differential recognition of different quadruplex surfaces with chemically diverse set of ligands. We have previously reported with the human telomeric G-quadruplex DNA that a combination of stacking and groove binding moieties can lead up to 1,000-fold binding affinity enhancement in the G-quadruplex recognition process. We have extended these studies by probing a well-resolved G-quadruplex DNA structure derived from the *Oxytricha Nova* telomeric DNA. From our studies, the following conclusions can be drawn:

(1) *BQQ-neomycin is the best Oxytricha Nova G-quadruplex binder of all neomycin-intercalators studied in this work*. The DC_50_ values obtained from FID titrations show that BQQ-neomycin is best in displacing the fluorescent probe bound to the G-quadruplex. The other neomycin-intercalator conjugates are at least 10 times better in displacing half of the fluorescent probe bound to these quadruplexes than neomycin. Hence conjugation of intercalators to neomycin improves their G-quadruplex binding. (2) *BQQ-neomycin binds Oxytricha Nova G-quadruplex DNA with 1:1 binding stoichiometry*. Both fluorescence and CD spectroscopy detected experiments confirm that BQQ-neomycin binds with the G-quadruplex in a 1:1 ligand to quadruplex ratio. The 1:1 binding stoichiometry is indicative of a single unique site for BQQ-neomycin complexation (3) *BQQ-neomycin binds with nearly 100-fold higher binding affinity than neomycin*. Scatchard analysis of the FID titration showed an association constant *K*_a_ = (1.01 ± 0.03) × 10^7^ M^−1^ which is 100-fold higher than neomycin. This result shows that conjugation of two DNA binding moieties can lead to higher affinity G-quadruplex ligands. Comparison with the rRNA A-site binding showed that the BQQ-neomycin's affinity is lower than that of the quadruplex (4) *The binding mode of BQQ-neomycin is strikingly different than neomycin or BQQ-Cl alone*. The CD experiments showed that binding of either neomycin or BQQ-Cl brings negligible change in the CD signal. However, when BQQ-neomycin was complexed with the G-quadruplex, a large increase in CD signal was noticed indicating significant binding. However, no induced CD change was observed in the absorption region of BQQ, which suggests that the BQQ interaction does not involve stacking with the guanine bases. The remarkable 1:1 ligand to quadruplex binding shows a simultaneous binding of the two moieties at independent binding sites. (5) *BQQ-neomycin conjugate is prone to aggregation*. Thermal melting and UV-vis absorption studies clearly showed that BQQ-neomycin is prone to aggregate at concentrations 4 μM or above. Therefore, caution must be taken while interpreting thermal melting results and designing experiments that require higher ligand concentrations. (6) The *total surface area of the intercalator unit may have implications in stacking with guanine bases*. The intercalator unit with three fused aromatic rings was found to have stacking interactions with the guanine bases while larger ring sizes prefer to interact predominantly through van der Walls interactions. Altogether, these results show that using our existing knowledge of ligands that are known to bind to quadruplex DNAs, a more potent ligand can be developed by combining the two separate ligands in a single molecule. As shown in this manuscript, the two binding moieties, neomycin and BQQ, have moderate binding, but when combined as one molecule, they result in a ligand with a much higher affinity and more importantly-selectivity in binding.

## Data Availability Statement

All datasets generated for this study are included in the article/Supplementary Material.

## Author Contributions

DA planned the experiments, supervised the data collection and analysis, and edited the manuscript. LX synthesized the conjugates. KA and NR performed the biophysical experiments. YA performed the docking studies. NR wrote the manuscript.

### Conflict of Interest

The authors declare that the research was conducted in the absence of any commercial or financial relationships that could be construed as a potential conflict of interest.
