# Supplementary material for: Surface Dependent Dual Recognition of a G-quadruplex DNA With Neomycin-Intercalator Conjugates
Source: Front Chem. 2020 Feb 12;8:60. doi: 10.3389/fchem.2020.00060 (PMC7028757; doi:10.3389/fchem.2020.00060)
Supplement: Supplementary file 1 [file Data_Sheet_1.PDF]

Supporting Information

Surface Dependent Dual Recognition of a G-quadruplex DNA using Neomycin-Intercalator Conjugates

Nihar Ranjan[a,b], Katrine F. Andreasen[a], Yashaswina Arora[b], Liang Xue[a], and Dev P. Arya[a,*]

[a]*Laboratory of Bioorganic Chemistry, Department of Chemistry, Clemson University, South Carolina, SC, United States 29634*

[b]*Department of Medicinal Chemistry, National Institute of Pharmaceutical Education and Research (NIPER),Raebareli, New Transit Campus, Lucknow, Uttar Pradesh, India 226002*

Table of Contents

S.No.	Title	Pg. No.
1.	FID titrations of Oxytricha Nova quadruplex with anthraquinone-neomycin	3
2.	FID titrations of hairpin duplex DNA with anthraquinone-neomycin	4

1. FID titrations of Oxytricha Nova quadruplex with various ligands

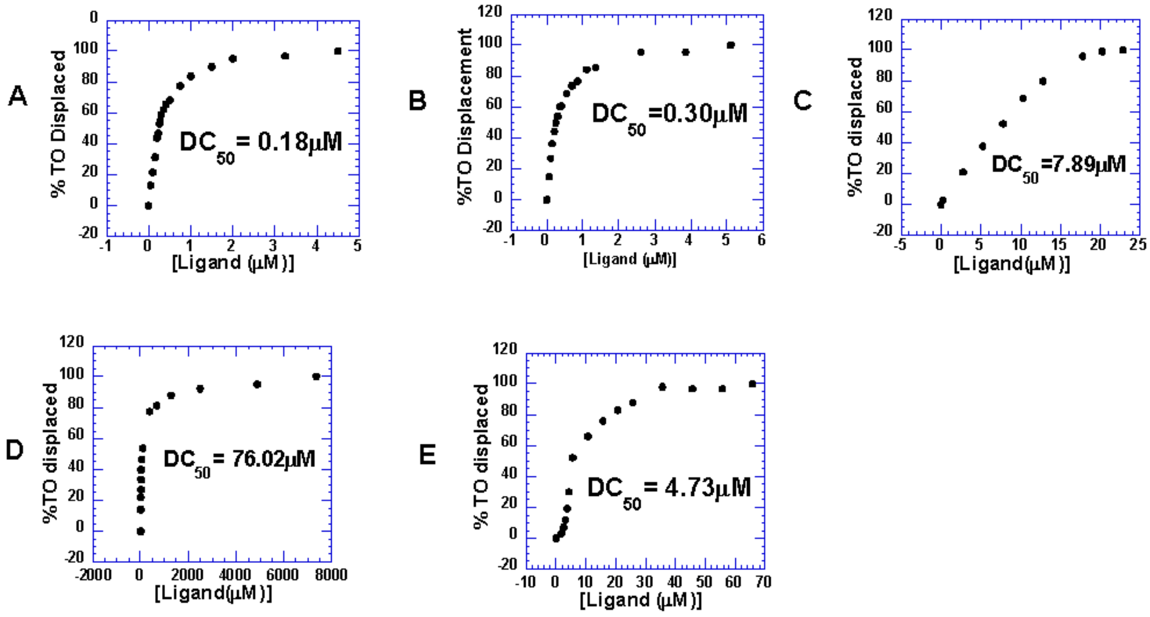

Figure S1: *TO displacement Plots with various ligands with oxytricha nova (GGGGTTTTGGGG) quadruplex: A) BQQ-neomcyin B) anthraquinone-neomycin C) naphthalenediimide-neomycin D) neomycin E) pyrene-neomycin*

2. FID titrations of hairpin duplex DNA with various ligands

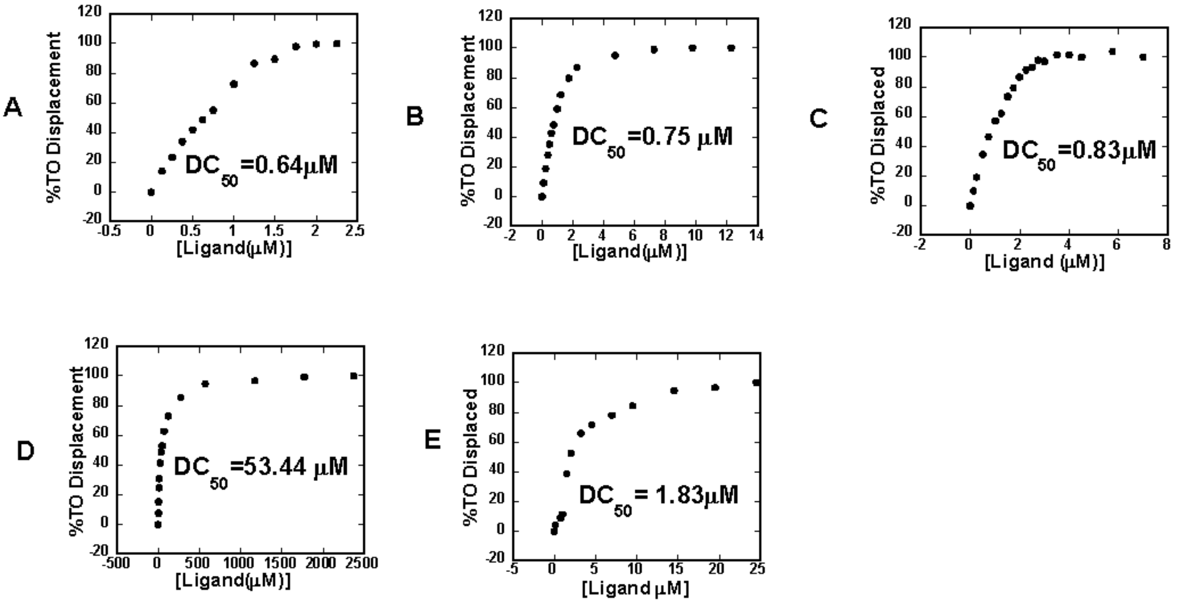

Figure S2: *TO displacement Plots with various ligands with hairpin duplex (AGGGGGTTTTTCCCCT): A) BQQ-neomcyin B) anthraquinone-neomycin C) naphthalenediimide-neomycin D) neomycin E) pyrene-neomycin*

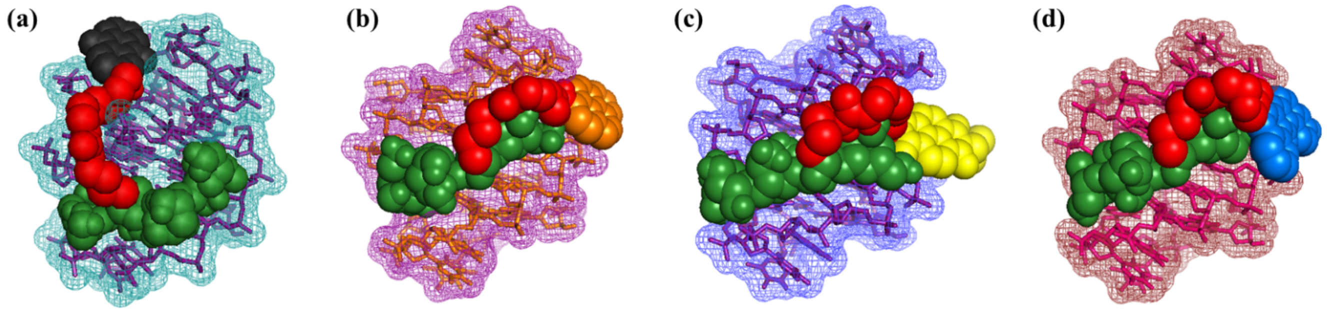

Figure S3. Lowest energy docked structures of various 'modified'neomycin-intercalator conjugates with *Oxytricha Nova* structure (a) Anthraquinone-neomycin (b) Pyrene-neomycin (c) Naphthalenediimide-neomycin (d) BQQ-neomycin. In each figure neomycin, linker and the Intercalator moieties have been shown in different colors. The modification is that in in these neomycin conjugates, the linker between the neomycin and the intercalators units is same as present in **compound 6**. The modification of linkers have been done in figures (a)-(c).
